# Integrated disease surveillance and response strategy for epidemic prone diseases at the primary health care (PHC) level in Oyo State, Nigeria: what do health care workers know and feel?

**DOI:** 10.11604/pamj.2018.31.19.15828

**Published:** 2018-09-07

**Authors:** Kola Ademola Jinadu, Akindele Olupelumi Adebiyi, Olubunmi Olutoyin Sekoni, Eniola Adetola Bamgboye

**Affiliations:** 1 Department of Community Medicine, University College Hospital, Ibadan, Nigeria; 2 Department of Epidemiology and Medical Statistics, Faculty of Public Health, University of Ibadan Nigeria

**Keywords:** Integrated disease surveillance and response, health care workers, epidemic-prone diseases

## Abstract

**Introduction:**

Effective diseases surveillance remains an important operational tool in countries with recurrent epidemic prone diseases (EPDs). In Nigeria, insufficient knowledge among Health Care Workers (HCWs) on Integrated Disease Strategy and Response Strategy (IDSR) have been documented. This study assessed knowledge and attitude of HCWs towards IDSR strategy for EPDs at the Primary Health Care (PHC) level in Oyo State, Nigeria.

**Methods:**

A cross-sectional facility based study using an interviewer-administered questionnaire was used to obtain information from 531 HCWs. In addition, 7 Key Informant Interviews was conducted. Discrete data were summarized as proportions while chi-square test was used to assess association between variables. A logistic regression model was used to assess predictors of knowledge of HCWs. All statistical significance was set at 5%.

**Results:**

Mean age of respondents was 42 ± 8.1 years with female preponderance (86.1%). Community Health Extension Workers (CHEWs) (36.9%) constituted the highest proportion of HCWs. About 70% and 90% of HCWs had good knowledge of EPDs and IDSR surveillance data flow respectively. Majority of HCWs 333(67.3%) knew how to use IDSR form 003 but less than 10% knew how to use other IDSR forms. The majority of HCWs {492(99.4%) and 345(69.7%)} agreed that reporting EPDs is necessary and IDSR tools are simple to use. Number of years post basic qualification was a predictor of HCWs' knowledge (AOR: 1.6; 95% CI: 1.0-2.3).

**Conclusion:**

This study showed poor knowledge on the use of IDSR forms although majority of HCWs had good knowledge and positive attitude towards IDSR strategy for EPDs. Thus, regular evaluation of health workers' knowledge and attitude towards IDSR strategy as a performance function of the surveillance system is recommended.

## Introduction

Epidemic prone diseases (EPDs) like viral haemorrhagic diseases, cholera and measles continue to pose major health risks to the health and welfare of human populations in developing countries including Nigeria [1]. These diseases have the potential to spread rapidly and affect a large number of people within a very short time period [2, 3]. This spread is being further worsened with increasing population mobility, globalization and increased risks of infectious diseases such as emerging and re-emerging diseases [4]. Effective disease surveillance remains one of the pillars of effective communicable disease control programme in most low and middle income countries [5]. The scope of a surveillance system is broad, from early warning systems for rapid response to communicable diseases, to planned response to chronic diseases, which `generally have a longer lag time between exposure and disease [5]. In response to the prevailing poor surveillance systems in the African region, the World Health Organization (WHO), African region adopted an improved surveillance system called “Integrated Disease Surveillance and Response (IDSR) strategy” as a regional strategy in 1998 [6-9]. The IDSR refers to a strategy and a tool that promotes rational use of resources by integrating and streamlining IDSR priority diseases (including EPDs) surveillance activities. Despite its importance, the IDSR strategy still suffers some setbacks especially in developing countries including Nigeria [10]. The weaknesses in the IDSR strategy in most countries had resulted in failures in detecting epidemics with an attendant spread of diseases and associated human suffering, and loss of lives [11]. The flow of information in the IDSR system in Nigeria is from the health facility to the Local Government Area (LGA), then to State Ministry of Health (SMOH) and finally to Federal Ministry of Health (FMOH). At the FMOH, data are collated and forwarded to the statistics division, analysis and feedback is carried out, as well as planning for appropriate intervention based upon the results of analysis [12].

In this regards, primary health care workers at the LGA level remains the mainstay of an effective and functional surveillance system. Apart from being a prerequisite for an effective surveillance system, Health Care Workers' (HCWs) knowledge of IDSR also enhances the performance of both technical and organizational tasks [13]. Similarly, knowledge amongst other factors has been identified to greatly influence HCWs' attitude towards reporting of EPDs [14]. Despite this, the knowledge of reporting requirements and responsibilities among HCWs has not been examined adequately as a cause of under-reporting [15]. In Nigeria, the collection, collation, analysis, interpretation and dissemination of data in healthcare facilities are often unsatisfactory, and this has been attributed partly to insufficient awareness and knowledge among HCWs on the importance of this process [16]. This is particularly important especially in the area of core IDSR activities like case definition, case detection, case registration, case reporting and data management. Moreover, only a few studies have been conducted on the evaluation of IDSR core functions in Nigeria (Edo, Kaduna, Anambra, Ekiti, and Osun) but none has been carried out in Oyo State especially with regards to the HCWs' knowledge of IDSR strategy for EPDs [17-20]. However, parts of Oyo State that share international borders with the Republic of Benin such as Iwajowa, Itesiwaju, Atisbo and Saki-west local government are at risk of trans-border transmission of epidemic prone diseases. In view of all these, this study will provide baseline empirical data needed for quality improvement in disease surveillance especially with regards to the knowledge of PHC workers on IDSR strategy for EPDs. This study determined the awareness and knowledge of HCWs about IDSR strategy for EPDs at the PHC level in Oyo State, Nigeria.

## Methods

The study was conducted in Oyo State, South-West Nigeria which has its capital in Ibadan [21]. It is bounded in the north by Kwara State, in the east by Osun State, in the south by Ogun State and in the west by Ogun State and partly by the Republic of Benin. Oyo State covers approximately an area of 28,454 square kilometers. It has an estimated population of about 5,580,894 and predominantly occupied by Yoruba people [21]. It is made up of three senatorial zones which are divided into 33 Local Governments Areas (LGAs). Oyo State has two teaching hospitals, 29 secondary health facilities, 11 specialist centers, 351 primary health facilities and 86 private health facilities [22]. A cross sectional facility-based study was conducted over a 4 week period (15^th^September-16 October, 2015). A mixed method approach was utilized consisting of a quantitative HCWs assessment tool and Key Informant Interviews (KIIs). Only HCWs involved in IDSR activities were enrolled in the study. These included Medical officers of Health, Nurses/Midwives, Disease Surveillance and Notification Officers (DSNOs), Laboratory scientists, Medical Record Officers, Community Health Officers (CHOs), Community Health Extension Workers (CHEWs) and Health Assistants. The Key Informant interviews (KIIs) were conducted among Medical Officers of Health (MOHs) and Disease Surveillance and Notification Officers (DSNOs) in each local government as they were identified as the gate keepers of any surveillance system. The sample size was determined using the Leslie Kish formula for calculating single proportions with a desired precision at 5% and an estimate of true proportion of health workers (70.9%) who do report notifiable diseases as documented in a similar study conducted in Northern Nigeria [23]. After making an adjustment for a non-response rate (10%) and design effect of 1.5, the minimum sample size was 528. However, 531 HCWs were interviewed in all. A cluster sampling technique was used to select the HCWs. Two Local Government Areas (LGAs) were selected from each of the three senatorial districts by balloting. An additional LGA was selected from Oyo south senatorial district based on proportional sampling as there are more LGAs in this district. In each LGA selected, all health care workers in all primary health care facilities who met the inclusion criteria were interviewed. Qualitative data was collected from six Medical Officers of Health (MOHs) and seven Disease Surveillance and Notification Officers (DSNOs) in the seven selected LGAs. (Only six MOHs and seven DSNOs were interviewed due to the absence of one of the MOHs). The KII guide used was adapted from a tool used by Sahal et al, to obtain staff views about the quality of communicable disease surveillance in Sudan [24]. Information was collected on extent of implementation of IDSR strategy for EPD, its feasibility, existing gaps and opportunities and resources needed for performing the core functions of the IDSR strategy. Quantitative data were collected with the use of tools adapted from the WHO/Centre for Disease Control (CDC) protocol for communicable disease surveillance system monitoring[25, 26].

Quantitative data collected include HCW's socio-demographic characteristics, knowledge of IDSR strategy and EPDs. The knowledge of the HCWs was assessed by asking questions on types of IDSR forms and their uses, epidemic prone diseases, and basic surveillance actions to be taken during epidemics. The types of IDSR forms asked for were IDSR 001A: used for immediate case based reporting of any notifiable disease; IDSR 001B: is the Laboratory Request form for notifiable diseases; IDSR 001C: -Line listing form which is a comprehensive summary of all suspected cases in an outbreak; IDSR 002-weekly reporting for 9 epidemic-prone diseases and public health events of international concern and the IDSR 003: which is the monthly reporting form for 41 priority diseases. In addition, knowledge of IDSR data flow and uses of IDSR forms was assessed by determining the proportion of HCWs with correct responses. In addition, knowledge of HCWs on EPDs was assessed by scoring correct responses as 1 and incorrect responses as 0. Using percentiles, a score of ≥75% was used as cut off mark for grouping the scores into good and poor knowledge of EPDs. The maximum obtainable score was 10 while the minimum obtainable score was 0. In addition, a cut off of 75% was chosen because a good surveillance system needs to be timely and complete and this depends on knowledge which should approach 100% [2]. Data were analyzed using SPSS software version 22. Descriptive statistics were derived and results were presented in frequency tables, charts and graphs as appropriate. Means and standard deviations were used to summarize quantitative continuous variables while Chi-square test was used to determine association between socio-demographic characteristics and categorical variables such as exposure to IDSR activities and knowledge of health care workers' on EPDs. Multivariate analysis using binary logistic regression was carried out to ascertain factors influencing knowledge of health care workers' on EPDs. The model was built based on logical factors that were significant at 10% and literature findings. Significance level for all statistical tests was set at 5%. Data from KIIs were transcribed and analyzed using the thematic approach to qualitative data analysis. The results of the KII were used to corroborate findings from quantitative data analysis Ethical approval for the study was sought and obtained from the Oyo State Research and Ethics Review Board while permission to conduct the study was obtained from the Chairman and MOH of each Local Government visited. A written informed consent was also obtained from the participants. Participants were interviewed privately and names HCWs or health facility were not documented to ensure confidentiality.

## Results

**Figure 1:**
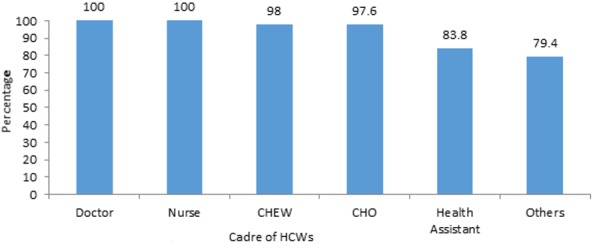
Awareness of IDSR strategy by designation of HCWs (others: lab scientists, medical record officers, pharmacist technicians)

**Healthcare workers' assessment:** Table 1 shows the socio-demographic characteristics of the health care workers. A total of 531 health care workers (HCWs) were interviewed with a mean age of 42 ± 8.1 years. Of these there were more females 457(86.1%) and urban residents 411(77.4%) while 64.8% were aged 40 years and above. A higher proportion of the respondents were married 492(92.7%) with 35(6.6%) unmarried, while only 4(0.8%) were widowed. Most of the respondents were Yoruba 521(98.1%) while 340(64%) and 91(36%) were Christians and Muslims respectively. Medical Doctors constituted only 1.1%, while a sizeable proportion were non doctors 325(98.9%). Of these non-doctors, CHEW constituted the highest proportion 196(36.9%). A majority of the HCWs 402(75.7%) had been in the present specialty for less than 10 years. A vast majority of the HCWs 495(93.2%) were aware of IDSR strategy for EPDs. Most of the HCWs were aware of form 003 341(69.1%) while 231(46.7%) and 51(10.3%) of them were aware of form 002 and form 001 respectively. However, only 11(2.2%) of HCWs were not aware of any form. All the doctors and nurses were aware of IDSR strategy while a sizeable number of other health workers were equally aware of this strategy. However, laboratory scientists, medical record officers and pharmacist technicians (79.4%) had the lowest level of awareness compared to other HCWs (Figure 1). The major source of information on IDSR strategy for EPDs was through HCWs (76.2 %%). This was followed by training/workshop (20.7%). An approximate equal proportion of the HCWs got their information through media and, relatives and friends. Majority of the HCWs 474(95.7%) correctly mentioned correct flow of surveillance data which is from health facility to local government, to state government and finally to federal government. Also, the majority of HCWs 333(67.3%) knew the use of form 003 while few, 49(9.9%), 42(8.5%), 11(2.2%), 6(1.2%) and 5(1.0%) of HCWs knew the use of form 002, 001, 001C, 001B and 001A respectively. A sizeable number of HCWs correctly mentioned the selected EPDs as EPDs. The EPD that a majority of HCWs correctly mentioned was cholera 480(97%) while yellow fever was least mentioned. Also, 79% of HCWs incorrectly listed tuberculosis as an EPD. (Table 2). In all, a sizeable proportion of HCWs 341 (68.9%) had good knowledge of the selected EPDs. These EPDs include cholera, shigella, measles, tuberculosis, viral haemorrhagic fever, leprosy, human influenza, yellow fever. Table 3 shows the associations between respondents' characteristics and knowledge of epidemic-prone diseases. A higher proportion of doctors/nurses 62(82.7) had good knowledge of EPDs compared with other HCWs 279(66.4%). With regards to level of education, the highest proportion of HCWs with good knowledge of EPDs was seen among those with university qualification 32(84.2%) followed by those with Polytechnics and equivalents 293(68.3%), and then those with up to secondary school qualifications 16(57.1%). A slightly higher proportion of HCWs 233(72.1%) with more than 10 years post academic qualification had good knowledge of EPDs compared to those with less than 10 years post basic qualification. The proportion of HCWs with good knowledge was slightly higher among those who have ever had formal training on core IDSR activities 120(75.0%) compared with those without any formal training 221(66.0%). All these associations were found to be statistically significant (p < 0.05). However, the associations between the age, gender, focal site, no of years in specialty and “ever been involved” in core IDSR functions and knowledge of EPDs were not statistically significant (p>0.05).

**Table 1:** Socio-demographic characteristics of the respondents

Variables	(N = 531)Frequency	Percentage
**Age(years)**		
<40	187	35.2
≥40	344	64.8
**Gender**		
Males	74	13.9
Females	457	86.1
**Highest level of education**		
Up to Secondary	44	8.3
Polytechnics and equivalents	449	84.5
University	38	7.2
**Religion**		
Christianity	340	64.0
Islam	191	36.0
Ethnicity		
Yoruba	521	98.1
Igbo	10	1.9
**Marital Status**		
Married	492	92.7
Single	35	6.6
Widowed	4	0.7
Location		
Urban	411	77.4
Rural	120	22.6
**Designation**		
Doctor	6	1.1
Nurse	69	13.0
CHO	84	15.9
CHEW	196	36.9
Health Assistants	142	26.7
Laboratory Technician	16	3.0
Medical Record Officer	16	3.0
Pharmacist Technician	2	0.4
**No of years since basic qualification**		
< 10 years	196	36.9
10 years and above	335	63.1
**Length of service in present specialty**		
< 10 years	402	75.7
≥ 10 years	129	24.3

**Table 2:** Knowledge of the respondents on IDSR related issues

Variables	N=495Frequency	Percentage
**Correctly stated pathway of IDSR data flow**		
Yes	474	95.7
No	21	4.3
**Correctly stated use of IDSR forms**		
Form 001	42	8.5
Form 001A	5	1.0
Form 001B	6	1.2
Form 001C	11	2.2
Form 002	49	9.9
Form 003	333	67.3
**Correctly listed the following as EPDs**		
Cholera	480	97.0
Measles	443	89.5
Viral haemorrhagic fevers	399	80.6
Human influenza fever caused by a new subtype	385	77.8
Diarrhoea with blood	366	73.9
Yellow fever	303	61.4
**Incorrectly listed the following as EPDs**		
Tuberculosis	390	78.8
Leprosy	212	42.8
HIV	212	42.8
Cancer	74	14.9
**Overall knowledge of EPDs**		
Good knowledge	341	68.9
Poor knowledge	154	31.1

**Table 3:** Association between respondents’ characteristics and knowledge of epidemic-prone diseases

Variables	Knowledge of epidemic prone diseases		χ^2^	p-value
	**Good (n=341)**	**Poor(n=154)**		
	**N (%)**	**N (%)**		
**Age(years)**				
≤39	109(64.9)	59(35.1)	1.91	0.17
≥40	232(70.9)	95(29.1)		
**Gender**				
Male	48(69.6)	21(30.4)	0.02	0.89
Female	293(68.8)	133(31.2)		
**Highest level of education**				
Up to Secondary	16(57.1)	12(42.9)		
Polytechnics and equivalents	293(68.3)	136(31.7)		
University	32(84.2)	6(15.8)	6.04	0.05
**Designation**				
Doctors/Nurses	62(82.7)	13(17.3)	7.83	0.01
*Other HCWs	279(66.4)	141(33.6)		
**Focal site for IDSR**				
Yes	190(72.5)	72(27.5)		
No	151(64.8)	82(35.2)	3.42	0.06
**No of years since basic qualification**				
< 10 years	108(62.8)	64(37.2)		
≥ 10 years	233(72.1)	90(27.9)	4.57	0.03
**No of years in specialty**				
< 10 year	260(70.1)	111(29.9)		
≥ 10 years	81(65.3)	43(34.7)	0.98	0.32
**Ever involved in formal training on core IDSR activity**				
Yes	120(75.0)	40(25.0)		
No	221(66.0)	114(34.0)	4.12	0.04
**Ever been involved in any core IDSR functions**				
Yes	323(69.9)	139(30.1)		
No	18(54.5)	15(45.5)	3.39	0.06

* CHOs, CHEWs, Laboratory scientist, medical record officers and pharmacist technicians, Health assistants

The predictor of knowledge of EPDs among HCWs is displayed in Table 4. The only predictor of good knowledge of EPDs among HCWs was number of years since post basic qualification. HCWs with more than 10 years since basic qualification are about two times more likely to have good knowledge of EPD compared to those with less than 10 years post basic educational qualification (O.R = 1.6; 95% CI = 1.03-2.4, p= 0.03). The majority of HCWs 492(99.4%) agreed that reporting EPDs is necessary although only 345(69.7%) agreed that the IDSR tools for reporting EPDs are simple to use. While about one-fifth of the respondents 64(21.9%) and a slightly above average 288(58.2%) respectively agreed that they were too busy to report EPDs and that reporting EPDs is time consuming, a sizeable number of HCWs 425(85.9%) agreed that a good reward system will increase their willingness to report EPDs. Despite the fact that the majority of the HCWs 482(97.4%) agreed that reporting EPDs is their public health responsibilities, about two-third of them 309(62.4%) still agreed that consents must be taken from patients before reporting EPDs. The qualitative findings showed that a number of factors influenced implementation of IDSR strategy for EPDs as highlighted by the majority of the respondents. The factors that negatively affect implementation of IDSR strategy for implementation of IDSR strategy for EPDs include poor funding, lack of adequate training and retraining of HCWs, paying too much attention to only focal sites, inadequate staff strength, and lack of logistic support e.g. Generators, computers, calculators, means of transportation, freezers, and IEC materials. A male respondent said *"we have so many challenges which include reduced staff strength, lack of logistic supports, and financial crisis. Also, people are retiring every day. Since six or seven years there was no appointment given to any health worker, we are short-staffed. Secondly, financial crisis and thirdly, the people working have overworked for so long and they are tired and sometimes forget disease that should be reported immediately. So the immediate reporting system may be a bit delayed"* (KII12). Similarly, a female respondent from a rural LGA stated *"lack of communication services is a major problem that sometimes delays immediate reporting of EPDs. When we suspect EPDs and there is no telecommunication service to report to the LGA DSNO, what do we do? Of course it will be delayed. Another problem is transportation problem. Our roads are bad and motorbikes occasionally ply our road. So, immediate reporting may be delayed"* (KII 4). Concerning the funding of IDSR strategy, majority of the respondents said that it is not well funded. They further stated that there is a budget line for surveillance activities but implementation still remains a major challenge. The financiers are state and local government while WHO and UNICEF also contribute their own quota. It was widely stated that state and local government have not been contributing towards surveillance activities despite having a separate budget line for it. A female respondent said *"poor funding is a major problem. Most times we spend our own money, use our own vehicles for surveillance activities. This is not fair at all"* (KII10). As regards the successes achieved in IDSR strategy, most of the respondents acknowledged that the introduction of this strategy has significantly improved index of suspicion of HCWs of EPDS. Another achievement mentioned was the prevention of outbreak, reduction of morbidity and mortality and conduct of regular training for HCWs. Factors highlighted as being responsible are mainly dedication on parts of the HCWs and also the support from WHO. A male respondent said *"if not for the support from WHO, we would not have recorded successes because state and local government have not been forthcoming with release of fund for surveillance activities"* (KII 11).

**Table 4:** Predictors of knowledge of epidemic-prone diseases among health care workers

Variables	Odd ratio	p-value	95% confidence-interval
**Highest level of education**			
Up to Secondary	1		
Polytechnics and equivalents	0.332	0.090	0.092 – 1.189
University	0.486	0.155	0.180– 1.314
**Designation**			
Doctors and Nurses	1		
Other HCWs	1.718	0.110	0.885- 3.334
**Working at focal sites for IDSR activities**			
Yes	1		
No	1.349	0.160	0.888-2.048
**No of years since basic qualification**			
< 10 years)	1		
≥ 10 years	1.591	*0.027	1.027 – 2.367
**No of years in specialty**			
< 10 years)	1		
≥ 10 years	1.545	0.107	0.911-2.620
**Ever had any formal training on core IDSR activity**			
Yes	1		
No	1.209	0.411	0.769-1.900
**Ever involved in core IDSR activities**			
Yes	1		
No	0.939	0.941	0.175-5.043

## Discussion

This study aimed to assess the knowledge and attitude of HCWs of the IDSR strategy for EPDs at the PHC level and the associated factors. A large proportion of HCWs in this study (93.2%) were aware of IDSR strategy for EPDs which is similar to what has been found by Nnebue et.al and Semevinatre et.al in Anambra State, Nigeria and Srilanka respectively [17, 27]. The similarity in awareness of IDSR strategy could be attributed to the fact that IDSR strategy for EPDs is part of routine data reporting activities of HCWs However, this study shows a higher proportion of those that knew the specific uses of IDSR form 003 compared to Nnebue et.al and Semevinatre et.al though the proportion of those that knew the specific uses of IDSR form 001 and 002 were lower in this study. Bawa et al, however in his study among primary health care workers in northern Nigeria showed a low IDSR awareness rate of 30.2% [23]. This low awareness can be attributed to probably lack of training in the study setting. The higher knowledge found on the specific uses of IDSR form 003 in our study can be attributed to the fact that IDSR form 003 are mostly available in all HFs and most HCWs are familiar with it because of its use for routine surveillance activities. HCWs' poor knowledge on the specific uses of IDSR forms 001 and 002 can be attributed to almost non-availability of these forms at the HFs. The higher proportion of those with knowledge of the correct flow of surveillance data found in this study is similar with that of Dairo et al. which put the proportion of HCWs with knowledge of data flow at 97.6% [20]. This similarity could be attributed to increased awareness and training during the recent Ebola outbreak in Nigeria. As regards the knowledge of HCWs on selected EPDs, this study shows that about one-third of the HCWs had poor knowledge of the selected EPDs which is in consonance with the finding of a study carried out by Tobin et al in Edo State on knowledge and attitude of HCWs towards Lassa fever in which he reported an overall poor knowledge of 38.9% [28]. Bawa et al also documented similar findings in Yobe State [23]. This similarity could be attributed to inadequate availability of standard case definitions for EPDs as well as poor training of HCWs on IDSR strategy for EPDs.

In this study, majority of doctors and nurses had good knowledge of EPDs compared with other cadres of HCWs which is comparable to a study conducted among primary health care workers by Aigbiremolen et al in Edo State where nurses and midwives (80.3%) had slightly better knowledge than other cadres of HCWs [29]. This further corroborates the fact that the higher the educational level of primary health care workers the better their knowledge and probably exposure to more training and workshops on EPDs. Greater number of years since basic qualification was also found to affect knowledge of EPDs. The results of this study demonstrated that greater number of years since basic qualification (≥ 10 years) was associated with good knowledge of EPDs. This finding is in consonance with that of Karim and Dilraj in Saudi Arabia which showed that greater number of years since basic qualification was associated with good knowledge of EPDs [30]. This similarity may be due to the fact that HCWs with a greater number of years since basic qualification would have acquired more experience in IDSR activities compared with those with less than 10 years since basic qualification. In addition, the result of this study which shows higher proportion of HCWs with good knowledge among those with previous training on IDSR activities is in agreement with the finding of Sow et al among HCWs in WHO-African region which also demonstrated that training of HCWs was associated with improved knowledge of HCWs about surveillance activities [31]. Concerning the attitude of HCWs towards reporting of EPDs, majority of HCWs in this study and that of Tan et al were of the opinion that reporting EPDs is necessary and that reporting EPDs is a public health responsibility of HCWs [32]. They also opined that good reward system for reporting and penalty for not reporting EPDs will increase HCWs' willingness to report EPDs. This similarity can be attributed to the fact that HCWs renumeration may be inconsistent and inadequate and often may not include allowances for additional duties. Contrary to the above findings, Karim and Dilraj in Saudi Arabia in their research among doctors found that most doctors were of the perception that it is useless to report EPDs [30]. This disparity in attitude was attributed to the fact that most doctors in that setting found notification forms too complicated and laborious to fill. On the other hand, most HCWs in this study agreed that reporting forms are simple to fill. Also, in contrast to the finding of this study, Karim et al in his study in Saudi Arabia showed a general poor attitude of HCWs towards reporting of EPDs [30]. This inconsistency could be attributed to the poor knowledge of HCWs about EPDs in the Karim et al study.

## Conclusion

This study concluded that a majority of HCWs were aware of the IDSR strategy, had good knowledge of EPDs and surveillance data flow. However, adequacy of knowledge on the use of the various IDSR forms was low. Therefore, more attention need to be devoted to regular evaluation of health workers knowledge and attitude to the IDSR strategy as a performance function of the surveillance system.

### What is known about this topic

- Primary health care workers are aware of the IDSR strategy;
- Primary health care workers are fully involved in the surveillance system using the IDSR strategy.

### What this study adds

- Knowledge of Epidemic Prone Diseases is not optimum among primary health care workers;
- Knowledge of IDSR 001A,B,B, 002 forms are not adequate among primary health care workers.

## Competing interests

The authors declare no competing interests.
